# Contribution of low birth weight to childhood undernutrition in India: evidence from the national family health survey 2019–2021

**DOI:** 10.1186/s12889-023-16160-2

**Published:** 2023-07-12

**Authors:** Arup Jana, Deepshikha Dey, Ranjita Ghosh

**Affiliations:** 1 Research Scholar, International Institute for Population Sciences, Mumbai, Maharashtra 400088 India; 2 MPhil., International Institute for Population Sciences, Mumbai, Maharashtra 400088 India; 3 PhD Scholar, Institute for Social and Economic Change, Karnataka, 560072 India

**Keywords:** Low birth weight, Stunting, Wasting, Underweight, India, NFHS 5

## Abstract

**Background:**

Infants born with low birth weight (LBW), i.e. less than 2500g, is considered an important factor of malnutrition in Asia. In India, research related to this issue is still neglected and limited. Evidence exists that a large number of child deaths occur in India due to maternal and child malnutrition-related complications. Moreover, it has been found that the cost of malnutrition in India results in a significant reduction of the country's Gross Domestic Product (GDP). Thus, in this current context, this study aims to explore the contribution of low birth weight to childhood undernutrition in India.

**Methods:**

The study used data from the 5^th^ round of the National Family Health Survey (NFHS-5), a large-scale survey conducted in India. The survey collected information from 176,843 mothers and 232,920 children. The study used the last birth information (last children born 5 years preceding the survey) due to the detailed availability of maternal care information. Univariate and bivariate analyses were conducted to determine the percentage distribution of outcome variables. Multivariate logistic regression was employed to examine the association between LBW and undernutrition (stunting, wasting, and underweight). The study also used the Fairlie decomposition analysis to estimate the contribution of LBW to undernutrition among Indian children.

**Results:**

The results show that childhood undernutrition was higher in states like Uttar Pradesh, Bihar, Jharkhand, Gujarat, and Maharashtra. The results of the logistic regression analysis show that infants born with low birth weight were more likely to be stunted (OR = 1.46; 95% CI: 1.41–1.50), wasted (OR = 1.33; 95% CI: 1.27–1.37), and underweight (OR = 1.76; 95% CI: 1.70–1.82) in their childhood compared to infants born without low birth weight. The findings from the decomposition analysis explained that approximately 14.8% of the difference in stunting, 10.4% in wasting, and 9.6% in underweight among children born with low birth weight after controlling for the individuals' selected characteristics.

**Conclusion:**

The findings suggest that LBW has a significant contribution to malnutrition. The study suggests that policymakers should prioritize strengthening maternal and child healthcare schemes, particularly focusing on antenatal and postnatal care, as well as kangaroo mother care at the grassroots level to reduce the burden of LBW and undernourished children.

## Introduction

In general, anthropometric measures are considered to be world's best indicators of physical well-being [1]. The better nutritional status of children is the desired outcome of development efforts in developing countries that emphasize human capital [2]. Inadequate food consumption leads to poor nutritional status, as do family planning, maternal health, poor healthcare, socio-economic, and environmental factors [3–8]. Globally, studies have shown a dramatically decreasing probability of child survival among malnourished children. Many of them die from diseases due to nutritional deficiencies [9, 10]. In the later phase of life, the surviving children suffer from diminishing learning and working capability, which is a human waste toll on the development of a nation, especially in developing countries like India [11].

Stunting, defined as height-for-age below -2 standard deviations (SD), wasting, indicated by weight-for-height below -2SD, and being underweight, characterized by weight-for-age below -2SD, are anthropometric measurements employed to evaluate the nutritional status of children [12, 13]. Childhood malnutrition has been proven to affect physical and mental health and significantly impede society's development by reducing labour productivity and increasing healthcare costs [14–16]. Every year, African and Asian countries lose 11% of their Gross Domestic Product (GDP) due to the burden of malnutrition, which costs more than the 2008–2010 financial crisis [17]. India lost 2.6% of its GDP due to the cost of micronutrient malnutrition [18]. Thus, reducing child malnutrition has become an important concern to achieve the SDGs targets on hunger and child health. The Indian Prime Minister announced the holistic nutrition or POSHAN Abhiyaan or National Nutrition Mission in 2018 to make India malnutrition-free by 2022, but it has failed to meet its goals [19].

According to the latest Global Hunger Index (GHI), which is based on total undernutrition and infant mortality, India ranked 107th out of 121 countries [20]. Moreover, having the highest number of stunted children is an emergency crisis of nutritional epidemiology that can be avoided by implementing interventions after identifying the major contributing factors of undernutrition. A study conducted in India found that protein intake and calorie intake are different determinants of undernutrition [6]. Further, wealth quintile, household dietary diversity, sanitation, history of diarrhoea, and vaccination were important predictors of undernutrition raised by studies conducted in different countries worldwide [3, 4, 21, 22]. Studies revealed that breastfeeding, maternal education, and income are also important predictors [23–25]. Environmental factors such as particulate matter 2.5 (PM_2.5_) and agriculture were found to be associated with childhood undernutrition [26, 27]. It can be observed that most previous studies have focused on the demographic and socio-economic determinants of the nutritional status of a child by neglecting the most significant factor—Low Birth Weight (LBW), which is an independent factor contributing to malnutrition among children, primarily in Asian and African countries [28].

Previous studies have revealed that malnutrition is much higher in children with LBW than in children without LBW [29–31]. According to the World Health Organisation (WHO), low birth weight babies are those who are born weighing less than 2500g [32]. Global estimates show that in 2012, approximately 15 million premature babies and more than 20 million LBW infants were born. An estimated 15% to 20% of all births worldwide are LBW, and the highest prevalence is observed in South Asian countries, where it is around 28% [32]. Although the prevalence of LBW in India reduced from 21% in 2006 to 18% in 2021, it is still higher than neighbouring countries such as Sri Lanka and Bhutan [33, 34], and the reduction rate is only 0.2% annually [35]. Therefore, the higher prevalence of LBW remains a persistent concern among decision-makers and researchers. However, according to causes of death statistics, approximately half of neonatal deaths occurred due to complications of LBW and premature birth [36]. Low birth weight babies are more susceptible to morbidities due to infection, feeding difficulties, temperature instability, pneumonia, cardiovascular disease, respiratory distress, and malnutrition [37–39]. In addition, LBW is highly correlated with different diseases, such as cough and diarrhoea [40, 41], which are the leading causes of childhood malnutrition in India [42, 43]. After growth falters during the neonatal period, infants fail to attain average height and weight, leading to wasting [44]. Evidence suggests that wasting in early life likely contributes to stunting in childhood [45]. Also, malnutrition during the foetal stage results in malnutrition throughout infancy, childhood, and adulthood [46]. Therefore, reducing the burden of LBW should be the first step in the fight against childhood malnutrition, which will indirectly reduce child mortality [47].

The Global Nutrition Report (GNR) reported that a $1 investment in a nutritional scheme could generate an economic return of $16 [20]. Thus, exploring the contribution of LBW to child undernutrition is necessary to increase the economic return by reducing undernutrition. In the Indian context, a connection between low birth weight and childhood undernutrition is missing, as previous studies have only explored the determinants of both [30, 48–53]. Moreover, in India, most studies on birth weight have been conducted using clinical data, and no work has been done at the national level using recent data. Therefore, this study aims to highlight the contribution of low birth weight to childhood undernutrition using a national-level cross-sectional dataset to fill this research gap. The study hypothesises that LBW is strongly associated with childhood undernutrition and has a significant contribution to stunting, wasting, and underweight. The findings may help implement evidence-based policy to achieve the targets of SDGs in India.

## Data and methods

The secondary data used for the analysis was taken from the recently conducted 5^th^ round of the National Family Health Survey (NFHS-5), 2019–21. It is a nationwide representative multi-round cross-sectional survey, equivalent to the DHS (Demographic and Health Surveys), that provides reproductive and child-related health information mainly. The 5^th^ round in the NFHS series provides information on population, health, and nutrition for India and each state and union territory. The survey used computer-assisted personal interviewing (CAPI), and data from 19 different languages were gathered using four survey questionnaires: household, women's, men, and biomarker. Due to the COVID-19 pandemic, the survey was conducted in two phases—Phase-I from June 17, 2019 to January 2, 2020, covering 17 states and 5 union territories (UTs), and Phase-II from January 30, 2020 to April 30, 2021, covering 11 states and 3 UTs. All COVID-19 protocols were followed during the data collection in the second phase. Men and women in the selected sample households between the age of 15–54 years were eligible for interviews. The NFHS-5 sample was designed to provide estimates of all key indicators at the national and state levels and for most key indicators at the district level (for all 707 districts in India as of March 31, 2017). India's overall sample size, which came to around 636,699 households, was determined by the number required to generate accurate indicator estimates for each district. With villages serving as the Primary Sampling Units (PSUs) in the first stage (picked with probability proportionate to size), the rural sample was selected using a two-stage sample design. In the second stage, 22 randomly chosen households were selected from each PSU. A two-stage sample design was also used in urban areas, with 22 randomly selected households in each Census Enumeration Block (CEB) in the second stage. After executing a thorough mapping and household listing operation in the selected first-stage units, households were selected for the second stage in both urban and rural areas. The multi-stage sampling was used in both phase of the survey. The nationwide survey covered 724,115 women in the reproductive age group of 15–49 years, and information about 232,920 children was collected from their mothers.

### Sample

**Fig. 1:**
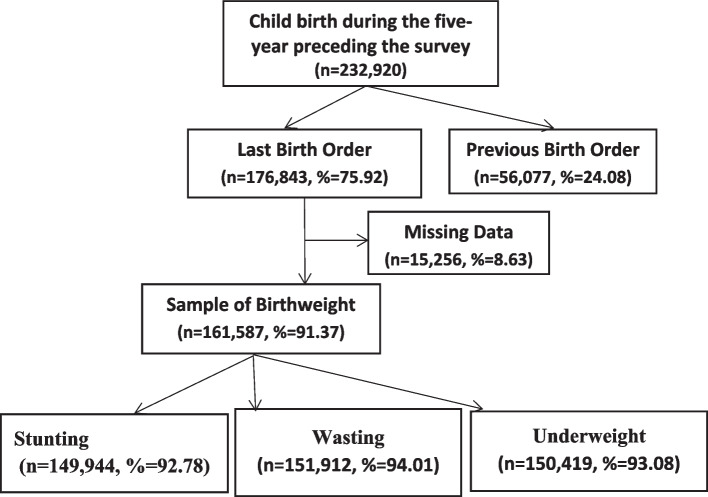
Schematic presentation of the sample used in the study

**Fig. 2:**
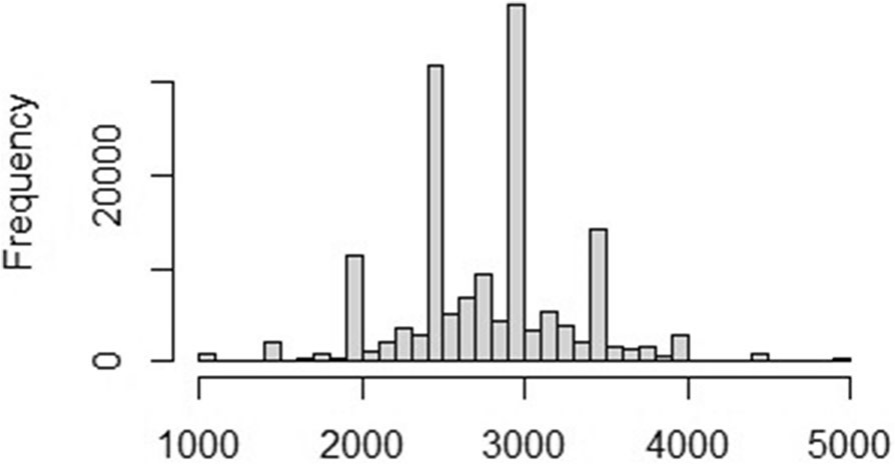
Histogram of birth weight in gram

Figure 1 shows the schematic presentation of the sample selection used for the analysis. Of the 232,920 births in the five years preceding the survey, 176,843 were the most recent (i.e., last-born) births. For the current analysis, only the last births were included because information on maternal healthcare was only available for a women's most recent pregnancy. Out of 176,843 infants, 15,256 infants had no birth weights or were missing, about 8% of the total sample, which does not significantly affect the results [54]. The histogram shows the heaping of birth weight around 3000g and the cut-off of LBW, 2500g (Fig. 2). In the study, half of the children's birth weights were not taken if they were born at home (Table 1). Missing birth weight data increases with the increasing number of children of an women. The missing birth weight was higher among mothers belonged to poor wealth quintiles, living in rural areas and mothers with no education than their counterparts. The flagged cases, means errors in the anthropometric measurement were 4,103, 9,504, and 888 for stunting, wasting, and underweight, respectively. In addition, respondents who refused or were not present were not included in the study. The final sample size was 149,925, 151,912, and 150,419 for height/age standard deviation, weight/height standard deviation, and weight/age standard deviation, respectively.

**Table 1:** Distribution of missing birthweight data by background characteristics

Background Characteristics	Missing LBW % (n)
Sex of child	
Male	9.91 (11,959)
Female	10.43 (11,706)
Home	50.44 (15,944)
Public hospital	3.76 (5,651)
Private hospital	4.06 (2,070)
Birth order	
1	6.54 (5,827)
2	8.41 (6,437)
3 & above	16.95 (11,401)
Mother's age at delivery	
< 20	10.06 (2,661)
20–24	9.25 (9,170)
25–29	9.82 (6,804)
30 & above	13.21 (5,030)
Mother's education	
Illiterate	20.07 (10,277)
Primary	13.93 (4,191)
Secondary	6.96 (8,339)
Higher	2.70 (858)
Place of residence	
Rural	11.22 (20,843)
Urban	5.98 (2,822)
Religion	
Hindu	8.49 (14,527)
Muslim	12.30 (4,122)
Others	17.70 (5,016)
Wealth status	
Poor	15.32 (18,056)
Non-Poor	4.88 (5,609)

### Outcome variables

The main outcome variables for the study were stunting, wasting, and underweight. The weight of children and adults was measured using the Seca 874 digital scale, while the height of adults and children aged 24–59 months was measured using the Seca 213 stadiometer. The Seca 417 infantometer was used to measure the recumbent length of children under two years or under 85 cm [35]. Verbal as well as written informed consent was obtained from all the participants. The informed consent for the children was taken from their parent or legal guardian. Nutritional status was assessed using three anthropometric indices: stunting (height-for-age), wasting (weight-for-height), and underweight (weight-for-age). Stunting refers to impaired growth and development that children experience due to poor nutrition, recurrent infections, and inadequate psychosocial stimulation. Wasting indicates recent and severe weight loss, usually caused by a lack of quality and quantity of food and/or frequent or chronic illnesses. Micronutrient deficiencies, which can cause growth and developmental delays, causes underweight [55]. WHO guidelines were used to create indices using standard deviation units (Z-scores) and the median of the reference population. Z-score less than minus 2 standard deviations from the median was used to define stunting, wasting, or underweight [56, 57]. In the study, the outcome variables were converted into dummy variables for the purpose of analysis. A value of 1 was assigned to indicate 'yes' and a value of 0 was assigned to indicate 'no'.

### Exposure variables

Low birth weight was considered as the main independent variable in the study. According to World Health Organization (WHO); if the weight at birth is less than 2500 g, then it is defined as LBW. It is known that undernutrition predicts childhood morbidity and controls nutritional status. The study included the child's characteristics such as sex of the child (male or female), birth order (1^st^, 2^nd^, 3^rd^ & above), age of the child (< 12 months, 12–23 months, 24–35 months, and 36 months & above) and breastfeeding practices (> 6 months and < 6 months). Maternal variables include the mother's education (illiterate, primary, secondary, and higher education) and the mother's age at birth (< 20 years, 20–24 years, 25–29 years, and 30 years & above). The National Family Health Survey-5 collected anthropometric measurements using biomarkers. Using that information, height of the mothers was categorized as < 145cm, 145-149cm, 150-154cm and > 154cm. Maternal healthcare variables such as antenatal visits, consumption IFA (Iron and Folic Acid) tablets and receiving benefits from ICDS (Integrated Child Development Services) centres or Anganwadi during pregnancy were taken in the analysis. A composite index of the household assets such as television and car, dwelling characteristics and other characteristics that related to wealth status was computed to measure the wealth status. Each household asset for which data was collected, assigned a weight or factor score through principal component analysis. The wealth quintile was recoded into three categories- poor, middle and rich. Religion was recoded into three categories- Hindu, Muslim, and Others. The households which collected drinking water from piped water, public taps, standpipes, tube wells, boreholes, protected dug wells and springs, rainwater, and community reverse osmosis (RO) plant was defined as improved source of drinking water. The households which had flush/pour flush toilets topiped sewer systems, septic tanks, pit latrines, or an unknown destination; ventilated improved pit (VIP)/biogas latrines; pit latrines with slabs; and twin pit/composting toilets and they did not share toilets with other households was defined as improved toilet facilities [35]. The community-level variable considered the place of the residence viz. rural or urban and regions namely North, Central, West, South and North-East.

### Statistical analysis

The prevalence of nutritional stunting, wasting, and underweight was estimated using the exposure variable of last birth or most recent birth of a woman. The spatial distribution of stunting, wasting, and underweight across states was examined in the study. Furthermore, the age-wise prevalence of nutritional status was compared between LBW and non-LBW children by plotting a line graph. A multivariate logistic regression model was used to adjust for socio-economic, demographic, and child-related characteristics to understand the association between low birth weight and nutritional status. For the analysis, the dichotomous variables of stunting (height-for-age), wasting (weight-for-height), and underweight (weight-for-age) were used as the dependent variable, and all states and union territories were included in the sample. The multivariate logistic regression model estimates the probability of an event depending on multiple sets of variables. The logistic regression model is defined as:

$$logit\left(p\right)=log\left(\frac p{1-p}\right)=\beta_0+\beta_1\ast x_1\dots\dots.+\beta_k\ast x_k+\varepsilon$$

Where ${\beta }_{0}$ is intercept and β1… βk are regression coefficients indicating the relative effect of a particular explanatory variable on the outcome, while $\varepsilon$ is an error term. In the study, the "svy" command in STATA was employed to account for the multi-stage sampling design.

Furthermore, we have used the extension of Binder Oaxaca decomposition given by Fairlie (2005), a non-linear decomposition technique appropriate for binary outcome variables. This technique decomposes the gap in the prevalence of diseases over the residence where the individual has spent most of their life, and we observe the percentage contribution of each of the attributable factors. Thus, a non-linear equation $Y=F\left(X\beta \right)$, can be decomposed as:

$${Y}^{r}-{Y}^{u}\left[\sum_{i=1}^{{N}^{r}}\frac{F\left({x}_{i}^{r}{\beta }^{r}\right)}{{N}^{r}}-\sum_{i=1}^{{N}^{u}}\frac{F\left({x}_{i}^{u}\right)}{{N}^{u}}\right]+\left[\sum_{i=1}^{{N}^{u}}\frac{F\left({x}_{i}^{u}{\beta }^{r}\right)}{{N}^{u}}-\sum_{i=1}^{{N}^{u}}\frac{F\left({\beta }^{u}\right)}{{N}^{u}}\right]$$

Where ${Y}^{r}$ and ${Y}^{u}$ represent the nutritional status among children with LBW and no LBW with samples ${N}^{r}$ and ${N}^{u}$, respectively. The first term in the equation represents t part of the gap due to group differences in the distributions of independent variables. The second term represents part due to differences in the group processes determining levels of Y and captures a portion of the group gap due to group differences in immeasurable or unobserved endowments. To identify the contribution of individual explanatory factors to the observed gap, we assume that the two-sample sizes are equal and that there is an exact match between the samples. Using coefficient estimates from a logit regression for a pooled sample β*, the independent contribution of *x*_*i*_*'s* to the group gap can then be expressed as:

$$\begin{array}{cc}{contribution}_{i}=\frac{1}{{N}^{u}}\sum_{j=1}^{{N}^{u}}F\left({a}^{*}+{x}_{ij}^{r}{\beta }_{i}^{*}+{x}_{j,j}^{r}{\beta }_{i,}\right)+F\left({a}^{*}+{x}_{ij}^{u}{\beta }_{i}^{*}+{x}_{i}^{r}{,}_{j}{\beta }_{i}^{*},\right),& \forall i \ne {i}^{,}\end{array}$$

The contribution of each variable to the gap is thus equal to the change in the average predicted probability from replacing nutritional status among LBW and No LBW children while holding the distributions of the other variables constant [58]. The analysis was performed by STATA version 16.1, R Version 4.1.1, and ArcMap version 10.8.

## Results

### Sample distribution

Table 2 shows the percentage distribution of the sample size used in the current study. In the study, 33% of the sample suffered from stunting, while 30% of the children were underweight. 19% of the sample was wasted, and 18% was LBW. Approximately 18% of the children's mothers were illiterate, and three out of four mothers followed the Hindu religion. About 73% of mothers received ICDS supplements during pregnancy, and 61% visited at least four times for antenatal care in India.

**Table 2:** Sample distribution of the study

Determinants	Percent	Sample (N)
Stunting (height-for-age)		
No (> -2 SD)	67.35	100,981
Yes (< -2SD)	32.65	48,944
Wasting (weight-for-height)		
No (> -2 SD)	81.44	123,714
Yes (< -2SD)	18.56	28,198
Under-weight (weight-for-age)		
No (> -2 SD)	70.16	105,531
Yes (< -2SD)	29.84	44,888
Low birth weight		
No	82.27	132,930
Yes	17.73	28,657
Sex of the child		
Male	53.73	86,824
Female	46.27	74,763
Age of the child (months)		
< 12	27.13	48,845
12–23	21.81	35,242
24–35	19.86	32,092
= > 36	31.20	50,408
Birth Order		
1	35.03	56,609
2	36.06	58,265
3 & above	28.91	46,713
Breastfeeding		
< 6 months	8.18	13,218
> 6 months	25.28	40,845
never breastfed	5.63	9,103
Still breastfeeding	60.91	98,421
Mother's age at delivery		
Below 20	9.14	14,773
20–24	40.52	65,483
25–29	32.33	52,233
30&above	18.01	29,098
Mother's height (cm)		
< 145	10.70	17,288
145–149	24.43	39,474
150–154	33.16	53,577
> 155	31.72	51,248
Mother's Education		
Illiterate	18.25	29,485
Primary	11.83	19,115
Secondary	53.89	87,084
Higher	16.03	25,903
Wealth Index		
Poor	45.72	73,892
Middle	20.21	32,663
Rich	34.06	55,032
Religion		
Hindu	74.68	120,666
Muslim	14.00	22,615
Others	11.33	18,306
Antenatal visit		
Less than 4	38.51	62,229
4 & more	61.49	99,358
IFA tablet		
Less than 100	47.98	69,170
100 & more	52.02	74,977
Received benefits from ICDS		
No	27.10	43,794
Yes	72.90	117,793
Place of residence		
Rural	77.69	125,532
Urban	22.69	36,055
Toilet facility		
Unimproved	27.25	44,025
Improved	72.75	117,561
Source of drinking water		
Unimproved	11.18	18,067
Improved	88.82	143,520
Region		
North	19.48	31,478
Central	24.41	37,440
East	18.07	29,198
North East	14.57	23,537
West	9.58	15,477
South	13.90	22,457
Survey phase		
Phase I	52.47	92,791
Phase II	47.53	84,052

### Spatial distribution of low birth weight and undernutrition in India

**Fig. 3:**
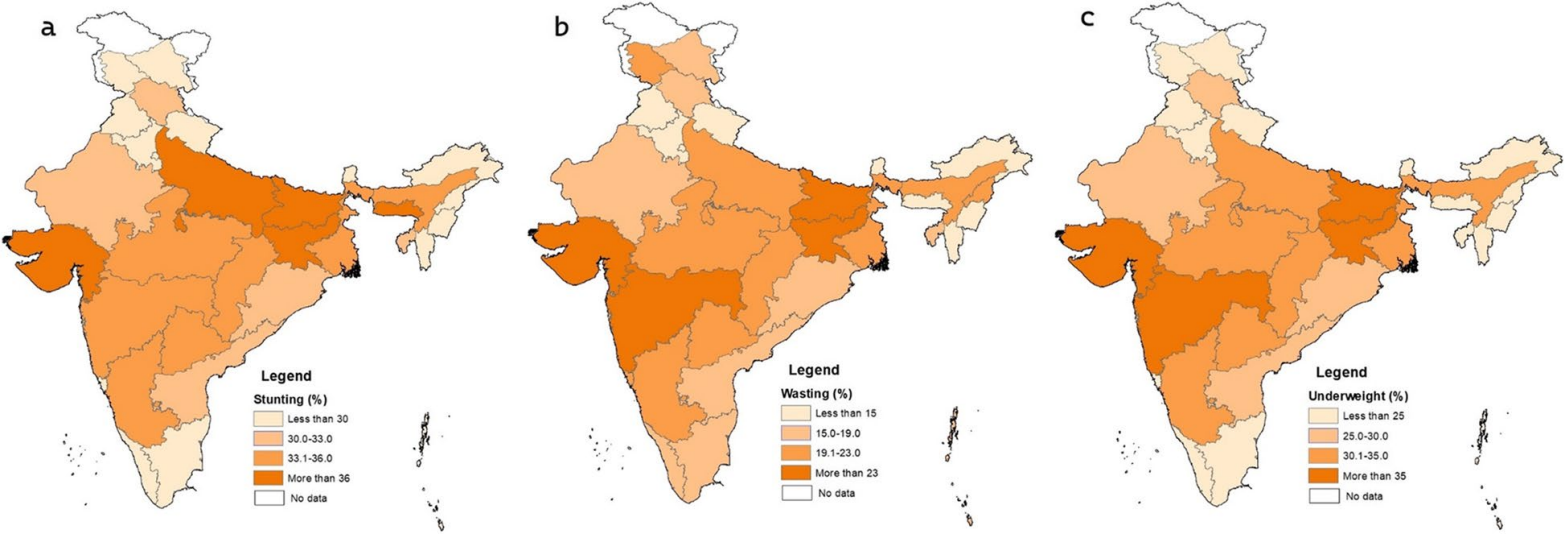
Spatial distribution of stunting (a), Wasting (b) and Underweight (c) in India, 2019–21

The results (Fig. 3) show that the prevalence of stunting was highest in the state of Meghalaya (40%), which is much higher than the national level prevalence of 33%. The state of Dadra & Nagar Haveli ranked second (Table 3), followed by Gujarat, Bihar, Uttar Pradesh, and Jharkhand. On the other hand, the percentage of wasting ranged from 25% in Maharashtra to 9% in Chandigarh. One-fourth of children suffered from wasting in Gujarat, Bihar, and Jharkhand. The severity of underweight incidence was highest in Bihar, which was around 39% and least in Mizoram with 12% of underweight. Additionally, states like Jharkhand, Dadra & Nagar Haveli, Gujarat, Maharashtra, and Madhya Pradesh had a higher percentage of underweight children. However, the national prevalence of LBW was 18%, and was higher in states like Punjab (22%), Delhi (21%), Madhya Pradesh (20%), Uttar Pradesh (20%), and Haryana (20%).

**Table 3:** State-wise percentage distribution of low birth weight, stunting, wasting, and underweight in India

**State**	**Stunting (**height-for-age < -2SD)	**Wasting (**weight-for-height < -2SD)	**Under weight (**weight-for-age < -2SD)	**Low birth Weight (< 2500g)**
Jammu & Kashmir	26.05	18.32	22.49	10.48
Himachal Pradesh	29.78	17.11	25.67	15.08
Punjab	22.84	10.27	16.02	21.84
Chandigarh	23.98	8.58	22.93	16.97
Uttarakhand	23.21	13.31	18.87	17.43
Haryana	25.89	12.17	20.89	19.89
NCT Of Delhi	28.91	11.56	21.28	21.39
Rajasthan	30.08	16.74	27.12	17.28
Uttar Pradesh	35.93	17.90	31.23	20.01
Bihar	37.28	23.71	38.47	16.84
Sikkim	22.35	11.68	13.02	9.31
Arunachal Pradesh	26.3	12.65	14.82	10.39
Nagaland	29.11	18.5	24.85	3.84
Manipur	21.42	9.41	12.00	7.30
Mizoram	26.16	10.05	11.52	4.03
Tripura	31.29	16.02	24.06	19.52
Meghalaya	39.67	14.17	24.60	11.90
Assam	33.13	20.51	31.98	15.34
West Bengal	32.59	19.84	31.86	18.75
Jharkhand	35.58	23.45	38.04	14.87
Odisha	29.97	18.15	29.08	18.38
Chhattisgarh	33.23	19.63	31.34	15.06
Madhya Pradesh	32.77	19.59	32.39	20.06
Gujarat	37.45	23.87	38.33	17.64
Dadra & Nagar Haveli	38.92	21.65	38.37	20.18
Maharashtra	33.40	24.92	35.16	19.39
Andhra Pradesh	29.51	16.07	28.80	15.23
Karnataka	33.41	18.17	32.11	15.01
Goa	23.17	20.52	22.44	13.29
Lakshadweep	30.55	15.96	25.6	9.89
Kerala	23.96	15.86	20.39	15.13
Tamil Nadu	25.12	14.52	22.04	15.82
Puducherry	18.94	11.49	15.21	12.73
Andaman & Nicobar Island	21.22	15.73	22.5	17.08
Telangana	32.22	20.40	31.71	12.94
Ladakh	27.89	16.39	19.90	10.58
India	32.55	19.18	30.84	17.73

### Age-wise prevalence of undernutrition by birth weight group

**Fig. 4:**
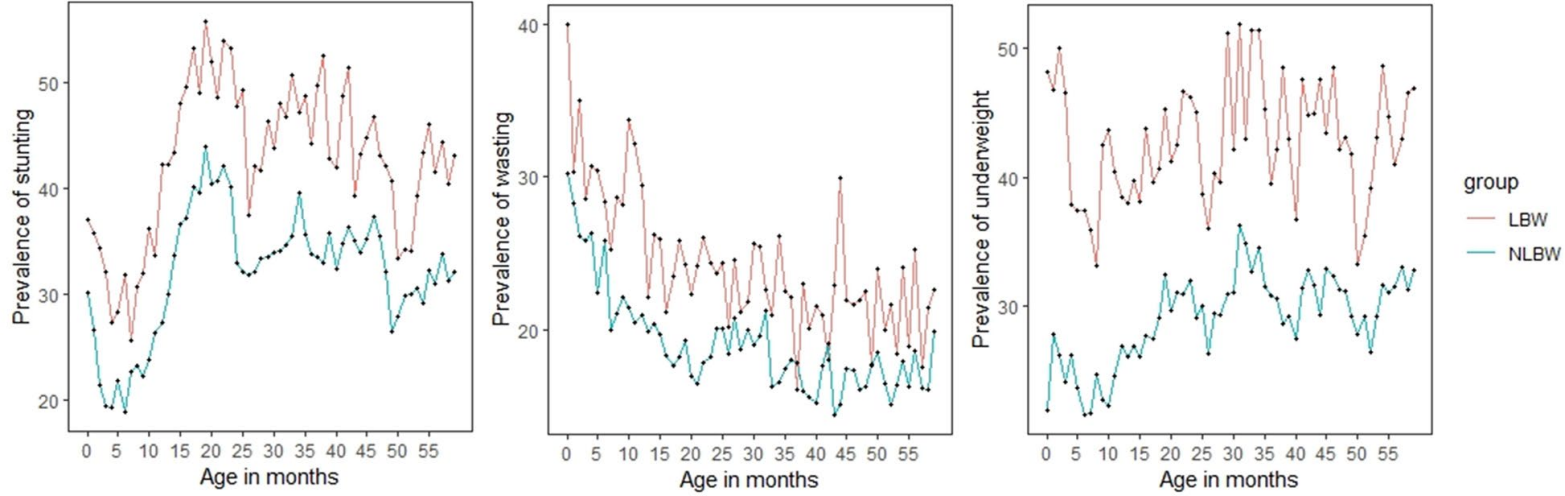
The prevalence of stunting, wasting, and underweight by birth weight from age 0–59 months India 2019–21. Note: LBW; low birth weight and NLBW; not low birth weight

Figure 4 shows that LBW infants had a higher prevalence of malnutrition than children with birth weights of  > 2500g. The percentage of stunting increased rapidly in the first 23 months of age, and then the curve flattened in later childhood. A large proportion of newborns were facing wasting at an early age; a decreasing slope was observed with the increase in their age. It was also found that the prevalence of wasting increased towards the end of childhood among LBW infants. The prevalence of underweight increased with the child's age, which was more visible among LBW babies.

### Association between low birth weight and undernutrition

Table 4 represents the association between LBW and malnutrition, namely stunting, wasting, and being underweight. Infants born with low birth weight were more likely to experience stunting (OR = 1.46; 95% CI: 1.41–1.50), wasting (OR = 1.33; 95% CI: 1.27–1.37), and underweight (OR = 1.76; 95% CI: 1.70–1.82) in their childhood compared to infants without low birth weight. The results also indicated that male children were more likely to be undernourished than female children. As expected, the probability of having a malnourished infant increased with decreasing levels of the mother's education and household wealth status. The most significant indicators of childhood malnutrition in India were children of younger mothers, mothers who were small in stature, and children who lived in urban areas. Additionally, as birth order increased, the likelihood of stunting and being underweight increased significantly, whereas the opposite was true for wasting. Children in the western region were more susceptible to stunting (OR = 1.19; 95% CI: 1.12–1.25) and underweight (OR = 1.56; 95% CI: 1.48–1.65). It is worth noting that mothers who received ICDS supplements during pregnancy had a higher probability of having an undernourished child.

**Table 4:** Logistic regression model to examine the association of low birth weight with stunting, wasting, and underweight, India

**Determinants**	**Stunting**			**Wasting**			**Underweight**		
	OR	95% CI		OR	95% CI		OR	95% CI	
		Lower	Upper		Lower	Upper		Lower	Upper
**Low Birth Weight**									
Yes	1.46***	1.41	1.50	1.33***	1.27	1.37	1.76***	1.70	1.82
No (Ref.)									
**Sex of the child**									
Male	1.16***	1.13	1.19	1.11***	1.08	1.14	1.18***	1.15	1.21
Female (Ref.)									
**Mother's education**									
Illiteracy	1.39***	1.32	1.48	1.26***	1.19	1.33	1.56***	1.47	1.63
Primary	1.35***	1.28	1.42	1.11***	1.05	1.18	1.42***	1.35	1.50
Secondary	1.15***	1.11	1.19	1.08***	1.03	1.13	1.20***	1.16	1.26
Higher (Ref.)									
**Wealth Index**									
Poor	1.46***	1.41	1.52	1.27***	1.21	1.32	1.57***	1.51	1.63
Middle	1.22***	1.17	1.27	1.10***	1.05	1.15	1.23***	1.18	1.27
Rich (Ref.)									
**Religion**									
Muslim	1.06***	1.03	1.11	1.14***	1.09	1.19	1.14***	1.09	1.18
Others	1.02	0.98	1.07	0.83***	0.79	0.87	0.83***	0.79	0.87
Hindu (Ref.)									
**Mother's age at delivery**									
below 20	1.33***	1.25	1.40	0.96	0.90	1.03	1.28***	1.21	1.36
20–24	1.20***	1.15	1.25	0.99	0.91	1.04	1.15***	1.11	1.20
25–29	1.06*	1.02	1.09	1.01	0.95	1.05	1.06**	1.02	1.10
Above 30 (Ref.)									
**Currently Breastfeeding**									
Yes	1.14***	1.1	1.17	1.14***	1.11	1.18	1.22***	1.19	1.26
No (Ref.)									
**Birth Order**									
2	1.15***	1.12	1.19	1.01	0.98	1.04	1.11***	1.08	1.14
3 & above	1.35***	1.30	1.39	0.99	0.95	1.03	1.26***	1.22	1.31
1 (Ref.)									
**Place of residence**									
Urban	1.01	0.98	1.04	0.95***	0.91	0.98	0.97	0.94	1.01
Rural (Ref.)									
**Age of the child (months)**									
< 12 (Ref.)									
12–23	2.06***	1.99	2.13	0.77***	0.74	0.79	1.22***	1.18	1.26
24–35	1.84***	1.77	1.91	0.82***	0.79	0.85	1.46***	1.41	1.52
= > 36	1.77***	1.69	1.84	0.73***	0.7	0.76	1.49***	1.43	1.55
**Mother's height (cm)**									
< 145 (Ref.)									
145–149	0.69***	0.66	0.72	1.02	0.97	1.07	0.73***	0.70	0.76
150–154	0.49***	0.47	0.52	0.99	0.94	1.04	0.57***	0.54	0.59
> 155	0.36***	0.34	0.37	0.92***	0.87	0.97	0.43***	0.42	0.45
**Antenatal visit**									
Less than 4 times (Ref.)									
4 & more times	1.01	0.98	1.03	0.99	0.96	1.02	1.00	0.97	1.03
**IFA tablet**									
Less than 100	1.03***	1.01	1.06	0.98	0.96	1.01	1.03**	1.01	1.05
100 & more									
**Received benefits from ICDS**									
No (Ref.)									
Yes	1.07***	1.04	1.1	1.04**	1.00	1.07	1.06***	1.03	1.09
Normal (Ref.)									
**Source of drinking water**									
Improved	1.10***	1.06	1.14	0.98	0.94	1.03	1.05***	1.01	1.09
Unimproved (Ref.)									
**Toilet Facilities**									
Unimproved	1.07***	1.04	1.1	1.03**	1.00	1.07	1.06***	1.03	1.09
Improved (Ref.)									
**Region**									
North	0.95**	0.91	0.99	0.84***	0.80	0.89	0.86***	0.83	0.90
East	0.83***	0.80	0.86	1.11***	1.06	1.16	1.01	0.97	1.05
North East	0.80***	0.76	0.85	0.84***	0.79	0.90	0.67***	0.63	0.71
West	1.19***	1.12	1.25	1.53***	1.44	1.63	1.56***	1.48	1.65
South	1.02	0.98	1.07	0.98	0.93	1.04	1.09***	1.04	1.15
Central (Ref.)									
**Phase of the survey**									
Phase I (Ref.)									
Phase II	0.93 ***	0.90	0.96	0.91***	0.88	0.95	0.95***	0.92	0.98

Note: (Ref.); Reference category, CI; confidence interval, OR; odds ratio, Significant level; ***p < 0.001, **p < 0.01, *p < 0.05

### Contribution of low birth weight to undernutrition

The results of the Fairlie decomposition analysis, examining the contribution of low birth weight to stunting, wasting, and underweight among children, are shown in Table 5. The study showed that the model could explain 14.8% of the difference in stunting, 10.4% in wasting, and 9.6% in underweight among children who were born with low birth weight after controlling for the selected socio-economic and demographic characteristics of the individuals.

**Table 5:** Decomposition model estimates the contribution of low birth weight to stunting, wasting, and underweight, India

**Independent Variables**	**Stunting**				**Wasting**				**Underweight**			
	Coef	*p* value	95% CI		Coef	*p* value	95% CI		Coef	*p* value	95% CI	
			Upper	Lower			Upper	Lower			Upper	Lower
Age of the child	0.001	0.000	0.001	0.001	-0.002	0.000	-0.003	-0.002	0.001	0.000	0.001	0.001
Place of residence	0.000	0.881	0.000	0.000	0.000	0.001	0.000	0.000	0.000	0.089	0.000	0.000
Mother's education	-0.003	0.000	-0.003	-0.002	-0.001	0.000	-0.001	-0.001	-0.004	0.000	-0.004	-0.003
Wealth index	-0.004	0.000	-0.005	-0.004	-0.002	0.000	-0.002	-0.001	-0.005	0.000	-0.006	-0.005
Mother's age at delivery	-0.009	0.000	-0.010	-0.009	-0.001	0.000	-0.001	-0.001	-0.007	0.000	-0.007	-0.006
Mother's height	-0.002	0.000	-0.002	-0.002	0.000	0.006	-0.001	0.000	-0.002	0.000	-0.002	-0.002
Sex of the child	0.002	0.000	0.001	0.002	0.001	0.000	0.001	0.001	0.002	0.000	0.002	0.002
Birth order	0.002	0.000	0.002	0.002	0.000	0.084	0.000	0.000	0.002	0.000	0.002	0.002
Phase of the survey	0.000	0.000	0.000	0.001	0.001	0.000	0.001	0.001	0.001	0.000	0.000	0.001
Number of observations	149925				151912				150419			
N of obs G = 0	125,281				126923				125673			
N of obs G = 0	24644				24989				24746			
Pr(Y! = 0G = 0) [a]	0.311				0.178				0.276			
Pr(Y! = 0G = 1) [b]	0.405				0.225				0.412			
Difference [a-b]	-0.093				-0.048				-0.136			
Total explained [c]	-0.014				-0.005				-0.013			
% Total contribution c/[a-b]*100	14.81				10.42				9.56			

** *p* < 0.01 *p < 0.005, *CI* Confidence interval, Dependent variable- Stunting (0 = No, 1 = Yes), Wasting (0 = No, 1 = Yes), Underweight (0 = No, 1 = Yes); Group Variable- low birth weight (0 = No, 1 = Yes)

## Discussion

The current study examined the impact of low birth weight on the nutritional status of children. Based on the analysis, Punjab has the highest prevalence of low birth weight, followed by Delhi, Madhya Pradesh, Uttar Pradesh, and Haryana. Stunting (33%), wasting (19%), and underweight (31%) are prevalent in India. Consistent with our hypothesis, the study finds a significant association between LBW and childhood stunting, wasting, and underweight. In India, LBW contributes to 14.8%, 10.4%, and 9.6% of stunting, wasting, and underweight cases, respectively. The study also identifies other factors such as the child's age, mother's height, household wealth status, mother's education, breastfeeding practices, and toilet facilities as key determinants of childhood undernutrition in India.

Previous studies have shown that the population of Delhi and the northern part of Uttar Pradesh is more exposed to ambient air pollution [59]. Moreover, a large volume of air pollution results from burning crop residue, a common practice among farmers in Punjab and Haryana [60, 61]. Previous evidence has suggested that maternal exposure to air pollution during pregnancy restricts fetal growth, indicating a strong relationship between LBW and air pollution [62, 63]. This may explain the high concentration of LBW in those regions. Furthermore, the states of Uttar Pradesh and Madhya Pradesh have the lowest maternal and child health care indicators. However, in Punjab and Haryana, one among three mothers received full antenatal care [55]. Although antenatal and postnatal care coverage has substantially improved in the central region of India, a large proportion of women still do not opt for institutional delivery or antenatal and postnatal check-ups [64, 65]. However, maternal and child health care indicators reflect the coverage of public health programs critical for LBW found in our study. These factors could be possible reasons for the high LBW prevalence in most of India's northern states.

Several previous studies have examined the socio-economic and demographic factors associated with childhood undernutrition. However, no study has been conducted in India to investigate the impact of LBW on childhood undernutrition, despite India having a higher prevalence of LBW compared to other developing nations such as Bhutan and Sri Lanka [33]. Children with low birth weight are at a higher risk of stunting and underweight up to the age of 23 months, while the percentage of wasting decreases with age. LBW increases the likelihood of reduced immune function, respiratory problems, and metabolic dysfunction, all directly associated with childhood illnesses, infections, and inadequate physical development [39, 40]. A study conducted in Zimbabwe found that LBW newborns develop more slowly than babies born with normal weight, and significant length differences persisted up to 12 months of age [66].

According to the life course perspective, undernutrition can begin during pregnancy and continue throughout life without given proper intervention [67]. The probability of giving birth to a malnourished child increases if the mother is undernourished, and if that child is a girl, it further increases the likelihood of intergenerational malnutrition. This cycle can be prevented by improving the nutritional status of newborn babies with LBW. The WHO introduced Kangaroo Mother Care (KMC), which helps to improve the physical health of LBW or premature babies by promoting skin-to-skin contact between newborns and mothers. It also recommends exclusive breastfeeding practices to protect babies from short-term or severe diseases found in the present study [68]. Health professionals can help mothers by providing modern technology and instructions to improve the health of LBW newborns. However, not all Indian mothers have access to this service, as the program has not yet been implemented nationwide [69]. Moreover, India can enhance birth weight by improving prenatal care facilities, educating women and mothers about proper food habits leading to proper nutrition, and increasing marital age and unnecessary caesarean sections, according to previous studies [70, 71].

Malnourished mothers have reduced protein and energy stores, smaller reproductive organs and less space for fetal development [72]. These elements impact both the fetus and the baby's growth through the placenta and the quantity and quality of breast milk, respectively. Additionally, genetics is predicted to significantly impact the relationship between a mother's height and that of her child [73]. According to research done in poorer and middle-income countries, a mother's small height is closely related to stunting and underweight in children [53], which is consistent with our findings.

The analysis of the current study revealed that the mother's wealth and education level were protective factors against child undernutrition. According to previous research, stunting, wasting, and being underweight have a substantial correlation with a mother's level of education and economic status [53, 55]. The knowledge about nutrition and health of the children rises with years of education of the mother. Additionally, the probability of using maternal and child health services rises with the mother's education level and household wealth status [74].

It is worth mentioning that antenatal care unitization, such as IFA supplementation or antenatal visit, did not significantly affect undernutrition in India. Surprisingly, we found a negative association between receiving supplements from Anganwadi during pregnancy and undernutrition among children. In India, most of the poor people go to Anganwadi for supplements, and about 30% of mothers have not received that benefit, which can be found in the dataset. We have not found a significant impact of that scheme on improving child nutrition, indicating the failure of ongoing maternal and child health programs in India. Thus, providing nutrition education programs might be useful to motivate people to receive that benefit by increasing awareness.

The study, for the first time, highlighted the contribution of low birth weight to child undernutrition in India using a large-scale survey. The estimates are robust as the present study was conducted using a large-scale survey, and therefore our findings can be generalized. Although the height and weight of mothers and their children were collected through anthropometric measurement, the weight of the child at the time of birth was reported by the mother's memory recall and health card. Thus, the study cannot ignore the possibility of biased reporting. The cross-sectional nature of the survey precludes establishing a causal relationship between LBW and child nutrition. Due to the unavailability of information on COVID, the study could not evaluate its impact on nutrition. The study was unable to control for premature birth due to the unavailability of gestational age information in the dataset.

## Conclusion

The study finds that low birth weight accounts for 14.8% of stunting, 10.42% of wasting, and 9.6% of underweight children. Furthermore, the child's age, mother's nutritional status, education, economic status and maternal healthcare of the household are also important contributors to undernutrition in India. Although many programmes are ongoing to control the nutrition status of children, the Indian government should focus more on preventing LBW since the nutritional events throughout the life cycle are crucial and cannot be neglected.

According to the study, it is suggested that the government should adopt and supervise certain special initiatives, such as KMC, to encourage breastfeeding and improve the development and nourishment of the LBW infants. The in-depth study also suggests that the high prevalence of undernutrition can be reduced with the improvement of healthcare services and the nutritional status of mothers in India.

## Data Availability

The study utilised the secondary data which is publicly available through https://dhsprogram.com/pubs/pdf/FR375/FR375.pdf

## Abbreviations

OR: Odds ratio
LBW: Low birth weight
KMC: Kangaroo mother care
BMI: Body mass index
NFHS: National family health survey
DHS: Demographic and health surveys
GDP: Gross domestic product
ICDS: Integrated child development service
